# Factors that determine first intubation attempt success in high-risk neonates

**DOI:** 10.1038/s41390-023-02831-8

**Published:** 2023-09-30

**Authors:** Donna Y. Chen, Bianca Devsam, Arun Sett, Elizabeth J. Perkins, Mitchell D. Johnson, David G. Tingay

**Affiliations:** 1 Neonatal Research, Murdoch Children’s Research Institute, Parkville, VIC Australia; 2 Department of Paediatrics, University of Melbourne, Melbourne, VIC Australia; 3 Neonatology, The Royal Children’s Hospital, Parkville, VIC Australia; 4 Department of Nursing, Melbourne School of Health Sciences, University of Melbourne, Melbourne, VIC Australia; 5 Department of Obstetrics and Gynaecology, University of Melbourne, Melbourne, VIC Australia; 6 Newborn Services, Joan Kirner Women’s and Children’s, Western Health, Melbourne, VIC Australia

## Abstract

**Background:**

Approximately 50% of all neonatal endotracheal intubation attempts are unsuccessful and associated with airway injury and cardiorespiratory instability. The aim of this study was to describe intubation practice at a high-risk Neonatal Intensive Care Unit (NICU) and identify factors associated with successful intubation at the first attempt.

**Methods:**

Retrospective cohort study of all infants requiring intubation within the Royal Children’s Hospital NICU over three years. Data was collected from the National Emergency Airway Registry for Neonates (NEAR4NEOS). Outcomes were number of attempts, level of operator training, equipment used, difficult airway grade, and clinical factors. Univariate and multivariate analysis were performed to determine factors independently associated with first attempt success.

**Results:**

Three hundred and sixty intubation courses, with 538 attempts, were identified. Two hundred and twenty-five (62.5%) were successful on first attempt, with similar rates at subsequent attempts. On multivariate analysis, increasing operator seniority increased the chance of first attempt success. Higher glottic airway grades were associated with lower chance of first attempt success, but neither a known difficult airway nor use of a stylet were associated with first attempt success.

**Conclusion:**

In a NICU with a high rate of difficult airways, operator experience rather than equipment was the greatest determinant of intubation success.

**Impact:**

- Neonatal intubation is a high-risk lifesaving procedure, and this is the first report of intubation practices at a quaternary surgical NICU that provides regional referral services for complex medical and surgical admissions.
- Our results showed that increasing operator seniority and lower glottic airway grades were associated with increased first attempt intubation success rates, while factors such as gestational age, weight, stylet use, and known history of difficult airway were not.
- Operator factors rather than equipment factors were the greatest determinants of first attempt success, highlighting the importance of team selection for neonatal intubations in a high-risk cohort of infants.

## Introduction

Endotracheal intubation is a lifesaving procedure frequently performed in Neonatal Intensive Care Units (NICUs).^1^ Due to the physiological and anatomical differences between neonatal, paediatric, and adult airways, successful neonatal intubations require specific skillsets and specialised knowledge.^2^ When compared to adult airways, neonatal airways are smaller in size with a shorter, narrower compact trachea, have relatively larger tongues, longer and more rigid epiglottis, and the larynx is positioned higher in the neck. Neonates also have smaller lung volumes and higher metabolic needs, resulting in less physiological reserve.^2^ Together, these pose an increased risk of tracheal intubation associated events (TIAEs), such as cardiorespiratory deterioration and airway injury.

First attempt success rates for intubations in the NICU are reported to be between 37 and 54%, and are influenced by operator experience and seniority, equipment used, presence of patient comorbidities, and location of intubation.^3–5^ Multiple intubation attempts can result in increased risk of airway injury including trauma to the glottic and subglottic mucosa, and formation of subglottic cysts which can lead to airway obstruction and stenosis.^6^ Experience and training are directly related to first attempt intubation success, with junior doctors having lower success rates than senior doctors.^7^ However, opportunities to gain experience are declining due to increasing use of non-invasive respiratory support and less invasive methods of surfactant administration without the need for an endotracheal tube (ETT), limiting intubation to the most critically unwell neonates.^7–9^ Most neonatal studies focus on preterm infants or intubations in the delivery room. Previous studies on neonatal intubation practices have focused on practices such as the use of video laryngoscopy and premedication amongst preterm infants and delivery room intubations; it is unclear whether the findings of these studies are translatable to other neonatal groups, such as the term neonate or those born with congenital anomalies.^1,10^

The NICU at The Royal Children’s Hospital (RCH) in Melbourne, Victoria, is a regional referral centre for neonatal airway disease and manages a heterogenous population of babies, including those requiring surgery and/or have specific high-risk medical needs. This creates both a different neonatal population but also an environment with a large variability of skills and experience in the management of difficult airways between operators. The National Emergency Airway Registry for Neonates (NEAR4NEOS) is an international registry that collects data on neonatal intubations across 19 different sites, including the RCH NICU. This database supports opportunities to explore factors and characteristics of all neonatal intubations to aid in providing evidence-based guidelines and instigate changes in current clinical practices to improve the quality of neonatal intubations. Thus, the aim of this study was to assess intubation events at RCH NICU and identify factors that are associated with successful intubation at the first attempt. We hypothesise that the success rate for first intubation attempts would be higher with increased operator seniority and lower with a known difficult airway.

## Methods

### Setting

This study was conducted in the NICU at RCH in Victoria, Australia. The RCH joined the NEAR4NEOS registry in February 2019 and was approved by the RCH Human Research Ethics Committee (HREA43754) in accordance with the National Health and Medical Research Council guidelines. The Children’s Hospital of Philadelphia (CHOP) is the primary sponsor and has approval from their Institutional Research Board for overall management of the NEAR4NEOS registry (eIRB 09-007253).

### Design

This was a retrospective cohort study using prospectively collected data on tracheal intubations and outcomes involving all neonates who were registrants in NEAR4NEOS at the RCH NICU over three years between February 2019 and March 2022. Intubation encounters were not included if the intubation occurred outside of the NICU. All intubations were performed using RCH intubation protocol which includes guidelines on staff roles, equipment, and difficult airway escalation. All data were collected using the NEAR4NEOS data forms in accordance with the NEAR4NEOS protocol. Details of the protocol, definitions, and data collection had been published in full previously.^1^ Deidentified intubation data were collected into a centralised online secure database hosted at CHOP. Each participating site had access to use data from their own population. Site participation requires a >95% compliance rate with intubation encounter, which is reviewed quarterly.

Within the NEAR4NEOS registry, an “intubation encounter” is defined as the entire intubation procedure for that infant, a “course” is defined as the intubation approach to airway management including the same device and premedication (but not operator) within an encounter. If device or premedication are changed, this defines a new course in that encounter. A single intubation attempt is defined as the beginning of the insertion of an airway device into the mouth or nose and ends when the airway device is removed or an ETT successfully placed by the same operator within a course. The successful placement of an ETT is defined as placement in the trachea; confirmed with direct visualisation, chest rise, auscultation, carbon dioxide detection, and/or chest radiography.^1^ First attempt success was defined as successful intubation by the first operator in the first attempt within that encounter.

### Outcome measures

The primary outcome was the number of attempts required to successfully place an endotracheal tube. Secondary outcomes consisted of patient factors including gestational age, weight, and airway grade, practice factors including use of premedication and stylet, and operator factors including training level and profession. A junior medical trainee was defined as a medical practitioner in specialist training with an average of 6–18 months of NICU experience, while a senior medical trainee was defined as a medical practitioner with an average of more than 18 months of NICU experience, including leadership and NICU subspeciality training. The order of operator seniority, arranged from least to most experienced, were as follows: Junior Trainee, Neonatal Nurse Practitioners (NNP), Senior Trainee, then Specialist Medical Staff. Adverse outcomes related to each intubation attempt (defined using the NEAR4NEOS protocol) were also recorded. Glottic airway grade was defined by the operator using the Cormak-Lehane grading system.^11^ The data entry form had images of each airway grade to reduce incorrect classification.

### Statistical analysis

Descriptive statistics were determined for each outcome by number of attempts. To simplify reporting, the number of attempts were defined as one, two, or three or more. The role of clinical, operator, equipment, and medication univariate factors, and success overall at each attempt were analysed using one-way ANOVA, chi-squared, or Fisher’s exact test (using first attempt success vs success at 2nd or subsequent attempt) as appropriate and reported as difference or odds ratio (OR) and 95% confidence interval (CI). Association between individual variables and intubation success were calculated with univariate logistic regression and presented as ORs (95% CIs). The multiple logistic regression model with variable selection was calculated by penalised LASSO regression (L1 penalty) after strongly correlated independent variables were excluded to mitigate multicollinearity. The corresponding ORs (95% CIs) from the multivariate model are reported. A *p*-value of <0.05 was considered significant and analysis was performed using PRISM V9.4 (Graphpad Software, San Diego, CA) or R statistical software (V4.1.2).

## Results

Data on 312 infants were collected from RCH NICU from 13 February 2019 to 2 March 2022. The characteristics of participating neonates are summarised in Table 1. The population was diverse, including both preterm and term infants, and recently born and older infants. Broadly, at time of intubation, infants were being managed on the NICU for a surgical problem (52.8%), sepsis (32.4%), and/or respiratory failure (43.6%). Respiratory failure (ventilation failure, apnoea, bradycardia, or hypoxia) were the most common reasons for intubation (77.5%). 40 infants (12.8%) had a known airway or craniofacial anomaly prior to intubation, and 27 infants (10.8%) were intubated for upper airway obstruction. Only 16 (6.2%) infants were intubated following unplanned extubation.

**Table 1:** Characteristics of participating infants.

Characteristics	*n* = 312
Gestational age at birth (weeks)^a^	35 (28,38)
Current age (days)	25 (7,49)
Birth weight (g)^b^	2140 (841,3030)
Current weight (g)	2670 (1645,3335)
Sex, *n* (%)	
Male	192 (61.5%)
Female	119 (38.1%)
Unknown	1 (0.3%)
Comorbidities, *n* (%)	
Sepsis	101 (32.4%)
Anatomical congenital anomaly requiring surgery	95 (30.4%)
Chronic respiratory failure	72 (23.1%)
Surgery/Procedure for acquired disorder	70 (22.4%)
Acute respiratory failure	64 (20.5%)
Congenital cardiac disease	55 (17.6%)
Airway or craniofacial anomaly	40 (12.8%)
Neurologic impairment	38 (12.2%)
Primary indication for intubation, *n* (%)	
Ventilation failure	78 (30.2%)
Frequent apnoea and bradycardia events	65 (25.2%)
Oxygen failure	55 (21.3%)
Procedure	48 (18.6%)
Upper airway obstruction	27 (10.8%)
Unstable haemodynamics	25 (9.7%)
Reintubation after unplanned extubation	16 (6.2%)
Other	71 (27.5%)

All data median (IQR) or number (%).

Missing data: ^a^*n* = 3, ^b^*n* = 1.

**Fig. 1:**
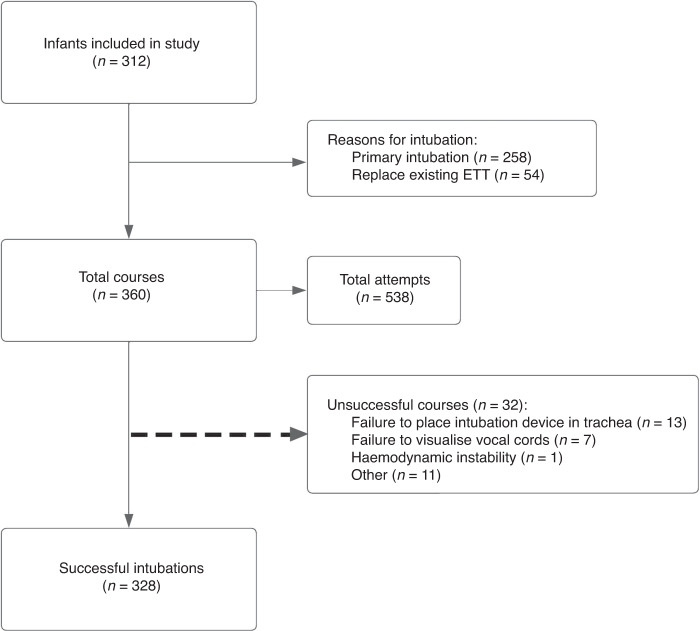
Study flow diagram. Summary of infant encounters, intubation courses, and attempts as per the Neonatal Emergency Airway Registry for Neonates definitions.

A total of 360 intubation courses were documented in the 312 infant intubation encounters. Of the 360 intubation courses, a total of 328 were successful in the NICU (91.1%). Within the 360 intubation courses, there were a total of the 538 intubation attempts (Fig. 1).

**Fig. 2:**
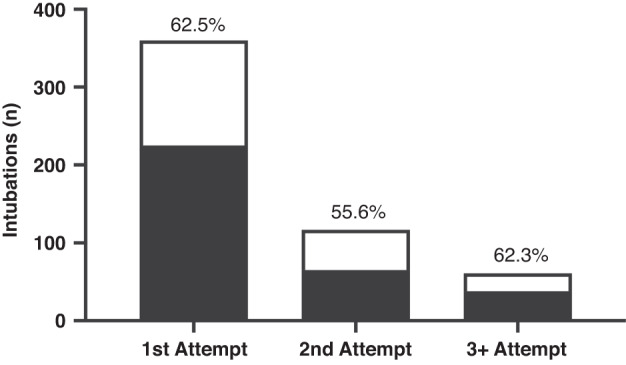
Intubation success by attempt. Black bars indicate the number of successful intubations at each attempt (total 328 successful intubations over 1–6 attempts). White bars indicate the total number of intubations at each attempt (538 total). Percentage of intubations that were successful at each attempt indicated in figure (*p* = 0.40; chi-square test).

A total of 225 (62.5%) intubation courses were successful on the first attempt and 65 (18.0%) on the second attempt. The proportion of successful intubations did not change between first, second, and subsequent attempts (Fig. 2; *p* = 0.40; chi-square test).

Most intubations were performed using a direct laryngoscope (492; 91.4%). The CMAC video laryngoscope was the next most commonly used intubation device (24; 4.5%). A laryngeal mask airway (LMA) was used in 7 intubations (1.3%). A stylet was used in 47.6% (256) of all intubation attempts but was not associated with increased chance of first attempt success (Table 2); OR (95% CI) 0.74 (0.48,1.14).

**Table 2:** Characteristics by number of attempts needed for successful intubation.

Number of attempts needed for successful intubation (*n* = 328)					
**Infant characteristics**	**Attempt 1** **(*n*** = **225)**	**Attempt 2** **(*n*** = **65)**	**Attempts 3+** **(*n*** = **38)**		
Gestational age, weeks (IQR)^a^	35 (28,38)	35 (27,38)	34.5 (28.5,38)		
Current age, d (IQR)^b^	24 (7,45)	29 (11,66)	26.5 (10,48)		
Birth weight, g (IQR)^c^	2244 (817,3030)	2150 (839.3,2962.3)	2127 (985.5,3162.8)		
Current weight, g (IQR)^d^	2680 (1585,3274)	2690 (2000,3600)	2988 (1808,3292.5)		
Sex^e^					
Male	143 (63.8%)	40 (61.5%)	23 (60.5%)		
Female	81 (36.2%)	25 (38.5%)	15 (39.5%)		
**Intubation characteristics**	**Total** **(*n*** = **328)**	**Attempt 1** **(*n*** = **225)**		**Attempt 2** **(*n*** = **65)**	**Attempts 3**+ **(*n*** = **38)**
Glottic airway grade					
Grade 1	229 (69.8%)	169 (75.1%)		42 (64.6%)	18 (47.4%)
Grade 2	72 (22.0%)	41 (18.2%)		17 (26.2%)	14 (36.8%)
Grade 3	11 (3.4%)	4 (1.8%)		4 (6.2%)	3 (7.9%)
Grade 4	2 (0.6%)	1 (0.4%)		0	1 (2.6%)
Grade 5	7 (2.1%)	7 (3.1%)		0	0
Glottic Grade 2 or more^f^	92 (25.6%)	53 (23.6%)		21 (32.3%)	18 (47.4%)
Endotracheal tube size					
2.5 mm	23 (7.0%)	12 (5.3%)		6 (9.2%)	5 (13.2%)
3.0 mm	131 (39.9%)	94 (41.8%)		24 (37.0%)	13 (34.2%)
3.5 mm	137 (41.8%)	92 (40.9%)		26 (40.0%)	19 (50.0%)
4.0 mm	29 (8.8%)	19 (8.4%)		9 (13.9%)	1 (2.6%)
Other parameters					
Stylet used	148 (45.1%)	95 (42.2%)		31 (47.7%)	22 (57.9%)
TIAEs					
Yes	44 (13.4%)	8 (3.6%)		15 (23.1%)	21 (55.3%)
No	284 (86.6%)	217 (96.4%)		50 (76.9%)	17 (44.7%)

All data median (IQR) or *n* (%). Data denote numbers for successful intubations only.

Number attempts (successful attempts): 3rd attempts 43 (26), 4th attempts 13 (8), 5th attempts 4 (3), 6th attempts 1 (1).

Missing data for gestational age, current age, birth weight, and current weight: (Attempt 1 and 2) ^a^*n* = 2, *n* = 2; (Attempt 2) ^b^*n* = 1; ^c^*n* = 2, ^d^*n* = 1.

Missing data for sex (Attempt 1): ^e^*n* = 1.

Missing data for glottic airway grade (Attempt 1, 2, and 3 + ): ^f^*n* = 3, *n* = 2, *n* = 2.

The glottic airway was a Grade 1 in 236 (65.6%) of all intubation courses, Grade 2 in 81 (22.5%), and Grade 3 or above in 28 (7.8%). The proportion of grade 2 or more airways increased from 23.6% of all successful first attempts to 47.4% of all successful intubations requiring 3 or more attempts (Table 2). A higher airway grade was associated with a lower chance of first attempt intubation success; OR 0.67 (0.52,0.88). A known history of a difficult airway was documented in 45 infants (14.4%) and was not associated with increased chance of first attempt success; OR 1.05 (0.92,1.22).

An ETT of size 3.0 or 3.5 mm was used in 81.1% of all attempts. As more attempts were required, smaller ETT sizes were used more frequently, with sizes 2.5 and 3.0 being used in 49% of all intubations requiring more than two attempts (Table 2). There was no correlation between ETT size and first attempt success; OR 0.66 (0.40,1.07). Patient factors like gestational age and weight of the infants at intubation were also not associated with first attempt intubation success; OR 0.99 (0.95,1.03) and 0.99 (0.99,1.00), respectively.

The occurrence of adverse events (TIAEs) increased with the number of attempts needed for successful intubation, from 3.6% for successful first attempts intubations to 35% when two or more attempts were required (Table 2). Immediate recognition of oesophageal intubation was the most commonly observed TIAE, occurring in 21 (0.6%) of the intubation courses (Supplementary Table 3).

Premedication with opioids, atropine, and muscle relaxants were routinely used for most intubations (Table 3).

**Table 3:** Medication use by intubation.

	Total (*n* = 328)	Successful 1st Attempt (*n* = 225)	Successful 2nd Attempt (*n* = 65)	Successful 3+ Attempts (*n* = 38)	*P*-value
Any premedication used	292 (89.0%)	198 (88%)	57 (87.7%)	37 (97.4%)	0.22
Opioid used	268 (81.7%)	179 (79.6%)	55 (84.6%)	34 (89.5%)	0.27
Atropine used	253 (77.1%)	166 (73.8%)	53 (81.5%)	34 (89.5%)	0.07
Muscle relaxant used	275 (83.8%)	185 (82.2%)	55 (84.6%)	35 (92.1%)	0.30

All data number (%). All *p*-values: chi-square test.

**Fig. 3:**
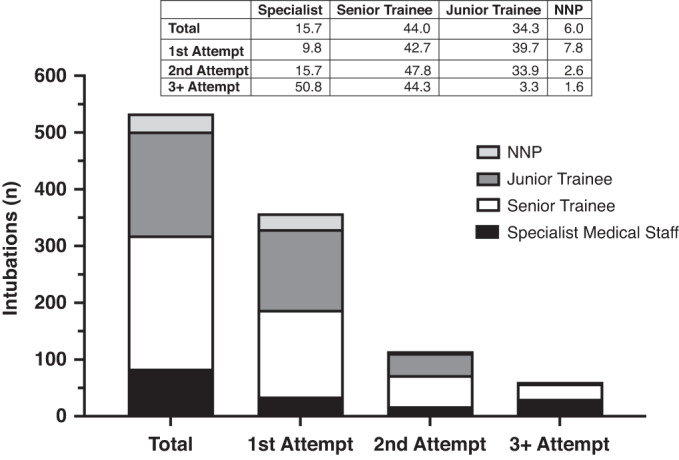
Skill level of operator at each intubation attempt. Black bars represent Specialist Medical Staff, white Senior Trainee, dark grey Junior Trainee, and light grey Neonatal Nurse Practitioner (NNP). Table represents the percentage (%) of each skill level by intubation attempt.

Overall, Senior Trainees conducted the most intubations (44.0% of attempts), followed by Junior Trainees (34.3%; Fig. 3). The proportion of intubations conducted by a Senior Trainee at each attempt was similar (42.7–47.8%). In contrast, Specialist Medical Staff performed 50.8% of all 3rd and subsequent attempts, but only 9.8% of first attempts. The reverse pattern occurred for Junior Trainees, decreasing from 34.3% of first attempts to only 3.3% of 3rd and subsequent attempts (*p* < 0.0001; chi-square test). Although NNPs performed the least number of intubation attempts (6.0%), they were most likely to be successful on the first attempt (*p* = 0.006; chi-square test). Higher operator seniority was associated with increased chance of first attempt intubation success; OR 1.66 (1.21,2.29).

Amongst the medical trainees, 88.0% of intubations were performed by physician trainees, with the rest performed by anaesthetic trainees. This decreased from 90% at first attempt to 88% and 69% for second attempt and 3+ attempts respectively (*p* = 0.003; chi-square test). Proportion of overall successful intubations were similar between the paediatric/neonatal and anaesthetic specialty groups; 62.1% and 62.3% respectively (Supplementary Table 1).

In a multivariate logistic regression analysis, higher operator seniority was independently associated with increased likelihood of achieving first intubation attempt success; OR 2.11 (1.66,2.67). Conversely, higher airway grade was associated with decreased chance of first attempt success; OR 0.58 (0.43,0.77), consistent with the univariate analysis results. The use of a stylet or a known history of difficult airway were not associated with first attempt success; OR 0.74 (0.48,1.14) and 1.08 (0.93,1.26) respectively.

## Discussion

This retrospective cohort study evaluated the current neonatal intubation practice in a quaternary referral teaching hospital NICU (RCH) and examined possible associations between clinical variables and first attempt intubation success rates, including patient demographics, operator characteristics, equipment and medications used, and number of adverse events. To our knowledge, this is the first report of intubation practices in a quaternary surgical NICU offering a regional referral service for airway conditions. We found that 63% of first intubation attempts were successful. This incidence is higher compared with the first attempt success rates of 37–54% reported in other studies involving perinatal NICUs.^1,3,4,12^ This could be attributed to the difference in infant demographics, as it is likely that the infants in our study were larger in size and more mature. The higher success rate may also be at least partly due to the case-mix in our NICU which lacks an onsite maternity service and delivery room care. Thus, intubations occurred solely within the more controlled NICU environment. Higher operator seniority and lower glottic airway grades were associated with increased first attempt success rates, and other patient factors such as gestational age, weight, and known history of difficult airway were not. We identified that operator selection rather than equipment selection was the greatest determinant of first attempt intubation success.

Our study found that the most junior operators (Junior Trainees) had the lowest overall intubation success rate (55%), which is expected since intubation is a learned practical skill. We also found that increased operator seniority was independently associated with increased first attempt success, consistent with findings from a similar study by Johnston et al.^13^ which reviewed 2608 neonatal intubations and found that advancing physician training levels were associated with increased first attempt success rates. The disparity between skill level is important due to the declining use of invasive ventilation requiring an ETT in neonates globally. A study by Leone at al.^5^ found that residents at a single academic centre were performing an average of 26 fewer intubation attempts during their training compared to residents 8 years prior. There are several reasons for this, including increased use of effective non-invasive ventilation, greater uptake of antenatal corticosteroids, less invasive surfactant techniques, increased requirement for senior staff to be present in the NICU, and the reduced working hours of junior doctors.^7,14–16^ However, as our study demonstrated, it remains critically important that proficiency in neonatal intubations is maintained amongst junior operators given that medical trainees still performed the majority of intubations.^4,13^ Simulation-based intubation training courses may help to bridge this gap of reduced real-life opportunities.^17,18^

Interestingly, we found that video laryngoscopy was rarely used for neonatal intubations (6%) despite the RCH NICU being a referral centre for airway conditions. This frequency was much lower than those reported in other studies.^10,19^ The reasons for this are not clear, but we speculate that this may be attributed to unfamiliarity with the equipment amongst staff and availability of equipment (cleaning), as well as the opportunity to perform very high-risk intubations in theatre with multi-disciplinary collaboration. The use of video laryngoscopy for neonatal intubation in recent years has increased following potential benefits for training and efficacy identified in some studies.^20–23^ These benefits include the ability to provide a wider laryngeal view, projection of the views onto a monitor to help facilitate indirect visualisation of the airway and guided advancement of the ETT, and real-time supervision and feedback.^22^ Whilst Moussa et al.^19^ concluded that the use of video laryngoscope did not increase rates of first attempt success, its use was associated with decreased number of TIAEs. In a study on video laryngoscopy training for junior doctors, first attempt intubation success rates improved by 25% when instructors were able to guide the trainees using video laryngoscope screens.^22^ Whether increased use of video laryngoscopy would increase the rates of first pass intubation success in our NICU population warrants further investigation.

Patient characteristics such as existing comorbidities and varying airway anatomy are crucial aspects of assessing intubation difficulty prior to commencing the procedure.^24^ Our study found that reduced visibility of the glottis was strongly associated with decreased first intubation success rates. Of all the intubations successful at first attempt, 75% of infants had a glottic exposure grade of 1, indicating that all of the glottic structures were visible.^11^ Studies that explored indicators of difficult intubations in neonatal and paediatric intensive care units reported that anatomical factors such as micrognathia, limited mouth opening, limited neck extension, and short thyromental distance were more common in patients that were difficult to intubate, however its sensitivity and positive predictive values were suboptimal.^10,25^ Alternative tools are being explored, including the application of airway ultrasonography for the prediction of difficult airways and higher glottic airway grades in adults.^26,27^ Further studies could be conducted to assess the additional utility of these risk stratification tools in the neonatal population. A strength of our study is that it was conducted in a unit that specialises in neonatal airway disorders. Approximately 15% of infants intubated in our study were known to have a difficult airway prior to intubation. However, airway status is rarely known in neonates prior to intubation. Further analysis should be conducted on this small population to identify potential factors that could aid success when a difficult airway is unexpectedly found.

There are several limitations to this study. The generalisation of our data is limited by the single site and its retrospective design and is therefore subject to recall bias, and may be influenced by local variations in practice. The highly specialised nature of our site also limits generalisability of our conclusions to tertiary perinatal institutes. Onsite training sessions on NEAR4NEOS protocol were provided to all staff, a standardised data form and internal data verification was performed by a dedicated member of the research team. Furthermore, studies in the quaternary neonatal surgical population are not common and large multi-site prospective data is difficult to collect. There is potential bias in difficult airway assessments as these are self-reported by the operators, introducing selection and reporting bias to both glottic airway grades and history of known difficult airways. Infant anatomical features such as micrognathia, limited mouth opening, limited neck extension, and short thyromental distance can contribute to difficult intubation, however these incidences were low in our cohort therefore were not analysed. RCH is an academic, teaching hospital, therefore the cohort of paediatric and neonatal trainees, number of intubation opportunities, and intubation equipment availability may differ from perinatal NICUs, and conclusions may not be translatable outside of academic teaching institutions. Prior intubation experience and training was not documented, although it was assumed that anaesthetic trainees and Senior Trainees had prior experience, and Junior Trainees were not allowed to intubate unsupervised. Our study did not consider the number of years of experience of operators and the varying degrees of experience amongst trainees, rather grading based on allocated hospital role. This may have an impact on intubation success and may limit the generalisability of these success rates across the subcategories of providers, although a similar hierarchal system occurs in most NICUs. Future studies should evaluate clinical factors that could better predict airway grades of 2 or above to help develop strategies to improve safety of neonatal intubations.

## Conclusions

In this single-centre retrospective study of high-risk infants admitted to a quaternary referral centre, operator experience rather than equipment was the greatest determinant of first attempt intubation success. Prior intubation experience and known glottic airway grades may guide team selection at intubation and identification of neonates suitable for less experienced intubators.

### Supplementary Information

Supplementary Materials

## Data Availability

All data, including raw data used for all figures and analysis, and allocation of specific subjects in previous published material is available upon request to the corresponding author from three months following article publication to researchers who provide a methodologically sound proposal, with approval by an independent review committee (“learned intermediary”). Proposals should be directed to david.tingay@mcri.edu.au to gain access. Data requestors will need to sign a data access or material transfer agreement approved by MCRI.
