# Supplementary material for: Factors that determine first intubation attempt success in high-risk neonates
Source: Pediatr Res. 2023 Sep 30;95(3):729–35. doi: 10.1038/s41390-023-02831-8 (PMC10899101; doi:10.1038/s41390-023-02831-8)
Supplement: Supplementary file 1 — Supplementary Materials [file 41390_2023_2831_MOESM1_ESM.pdf]

Supplementary Materials

NEAR4NEOS definitions

1. Encounter: refers to complete sequence of events leading to a placement of an advanced airway.
2. Course: refers to one method or approach to secure an airway and one set of medications (including premedication and induction). Each course may include one or several "attempts" by one or several providers.
3. Attempt: is a single advanced airway manoeuvre (e.g. tracheal intubation, LMA placement), beginning with the insertion of a device, e.g. laryngoscope (or LMA device) into patient's mouth or nose, and ending when the device (laryngoscope), LMA or tube is removed.

Supplementary Table 1. Proportion of successful intubations by medical trainee speciality

Medical Trainee Speciality	Total	Attempt 1	Attempt 2	Attempt 3+
Paediatric/Neonatal	247/398 (62.1%)	188/292 (64.4%)	47/86 (54.7%)	12/20 (60.0%)
Anaesthetic	33/53 (62.3%)	19/32 (59.4%)	8/12 (66.7%)	6/9 (66.7%)

All data number of successful attempts by total number of attempts (% success).

Supplementary Table 2. Proportion of successful intubations by skill level

Skill level	Total	Attempt 1	Attempt 2	Attempt 3+
Specialist Medical Staff	47/84 (56.0%)	18/35 (51.4%)	10/18 (55.6%)	19/31 (61.3%)
Senior Trainee	150/235 (63.8%)	103/153 (67.3%)	30/55 (54.5%)	17/27 (63.0%)
Junior Trainee	100/183 (54.6%)	79/142 (55.6%)	20/39 (51.3%)	1/2 (50.0%)
NNP	28/32 (87.5%)	24/28 (85.7%)	3/3 (100.0%)	1/1 (100.0%)

All data number of successful attempts by total number of attempts (% success).
Missing data for overall intubation attempts (Total, Attempt 1, Attempt 2): n=4, n=2, n=2

Supplementary Table 3: Summary of Adverse TIAEs

TIAE	Total
Cardiac Arrest – patient died	1
Cardiac Arrest – patient survived	7
Mainstem intubation	1
Oesophageal intubation, immediate recognition	21
Vomit with aspiration	1
Vomit, No aspiration	3
Cardiac compressions (< 1 min)	8
Gum or dental trauma	1
Pneumothorax/Pneumomediastinum	1
Dysrhythmia (includes bradycardia < 60/min)	16
Pain/agitation, required addition medication and delay in intubation	2
Other	10
